# Supplementary material for: Benchmarking free energy calculations: Analysis of single and double mutations across two simulation software platforms for two protein systems
Source: PLoS One. 2026 Apr 3;21(4):e0335829. doi: 10.1371/journal.pone.0335829 (PMC13048485; doi:10.1371/journal.pone.0335829)
Supplement: S1 Table — The experimental values and previously reported values using Schrödinger or GROMACS alongside values calculated in this study using both platforms are provided for comparison. (PDF) [file pone.0335829.s001.pdf]

S1 Table: Folding free energy changes ($\Delta\Delta G$) in kcal/mol for 38 single mutants (SMs) of the S. nuclease protein. The experimental values and previously reported values using Schrödinger or GROMACS alongside values calculated in this study using both platforms are provided for comparison.

S. No.	SMs from S. nuclease	$\Delta\Delta G_{Exp}$ [1,2]	$\Delta\Delta G_{Schrödinger\ or\ GROMACS}$ (previously reported)[1,2]	$\Delta\Delta G_{GROMACS}$ (Calc.)	$\Delta\Delta G_{Schrödinger}$ (Calc.)	GROMACS (Calc.) Using MBAR
1	**T22C**	0.9	-0.35	0.56 ± 0.50	-0.18 ± 0.19	0.91 ± 0.03
2	**T22V**	0.9	1.05	1.08 ± 0.50	0.92 ± 0.17	1.27 ± 0.03
3	**V23L**	0.1	-0.34	-0.24 ± 0.65	-0.2 ± 0.12	-0.07 ± 0.02
4	**L25I**	1.7	1.72	1.18 ± 0.34	1.76 ± 0.09	1.26 ± 0.02
5	**T33V**	-0.4	-0.51	0.06 ± 0.18	-0.74 ± 0.08	0.03 ± 0.02
6	**T41C**	-0.6	-0.52	-0.51 ± 0.28	-0.68 ± 0.16	-0.32 ± 0.02
7	**T41I**	-0.7	-1.4	-1.08 ± 0.17	-1.83 ± 0.18	-1.06 ± 0.02
8	**T41S**	1.1	1.04	0.84 ± 0.49	1.21 ± 0.16	0.89 ± 0.02
9	**T41V**	-0.8	-1.69	-0.79 ± 0.25	-2.06 ± 0.14	-0.8 ± 0.03
10	**T44V**	-0.1	0.04	0.84 ± 0.64	0.21 ± 0.17	0.92 ± 0.05
11	**S59A**	-0.5	-0.73	-0.32 ± 0.25	-0.49 ± 0.14	-0.28 ± 0.02
12	**T62S**	2.1	1.09	0.56 ± 0.67	0.59 ± 0.16	0.45 ± 0.02
13	**T62V**	0.2	-2.43	-0.19 ± 0.37	-2.53 ± 0.11	-0.16 ± 0.02
14	**V66I**	1	-0.5	0.44 ± 0.19	-0.69 ± 0.11	0.45 ± 0.14
15	**I72L**	0.2	0.54	0.64 ± 0.16	0.38 ± 0.06	0.86 ± 0.62
16	**T82S**	0.7	0.92	0.83 ± 0.73	1.27 ± 0.81	0.61 ± 0.02

17	**I92V**	0.4	1.04	1.34 ± 0.26	0.57 ± 0.07	1.3 ± 0.02
18	**K116G**	-1	-2.88	-0.4 ± 0.61	-2.25 ± 0.17	-0.51 ± 0.03
19	**T120C**	1.7	1.62	0.88 ± 0.55	0.94 ± 0.22	1.25 ± 0.01
20	**T120S**	0.6	0.83	-0.04 ± 0.48	0.33 ± 0.53	0.42 ± 0.05
21	**T120V**	1.8	4.11	1.23 ± 0.59	3.69 ± 0.21	1.07 ± 0.03
22	**S128A**	-0.7	-2.01	-1.01 ± 0.23	-2.48 ± 0.10	-1 ± 0.04
23	**V66K**	7.5	0.6	7.59 ± 0.11	11.83 ± 0.80	7.58 ± 0.03
24	**L7A**	1.59	1.3	-0.17 ± 0.22	-0.06 ± 0.09	0.04 ± 0.02
25	**I15V**	0.81	-1.67	1.23 ± 0.24	0.65 ± 0.06	1.19 ± 0.08
26	**I18M**	0.5	1.93	-0.45 ± 0.49	-0.9 ± 0.09	-0.48 ± 0.02
27	**V23F**	2.31	0.67	0.69 ± 0.53	1.76 ± 0.13	0.58 ± 0.01
28	**T33S**	1	1.7	1.15 ± 0.44	1.08 ± 0.43	1.36 ± 0.02
29	**L37A**	1.69	0.72	1.41 ± 0.64	1.41 ± 0.16	1.42 ± 0.02
30	**T62A**	2.41	0.95	1.09 ± 0.49	-1.68 ± 0.14	0.74 ± 0.02
31	**V66L**	0.1	2.16	-1.46 ± 0.41	-3.6 ± 0.50	-1.34 ± 0.02
32	**A69T**	2.64	1.98	2.29 ± 0.36	3.23 ± 0.97	2.2 ± 0.04
33	**I72V**	1.78	1.84	1.53 ± 0.20	1.53 ± 0.08	1.46 ± 0.02
34	**G79S**	2.67	0.31	0.53 ± 0.65	2.45 ± 0.18	0.22 ± 0.02
35	**Y85A**	0.41	1.8	0.64 ± 0.32	1.23 ± 0.11	0.66 ± 0.02
36	**A90S**	1.91	-0.11	2.56 ± 0.25	3.32 ± 0.09	2.44 ± 0.01
37	**Y113A**	-0.03	1.46	0.48 ± 0.15	-0.38 ± 0.08	0.51 ± 0.01
38	**A130G**	1.12	6.69	1.05 ± 0.35	1.52 ± 0.06	1.2 ± 0.02

	RMSE			0.13	0.24	0.13

Pearson correlation for all 38 SMs	Pearson r	R^2
**Exp vs GROMACS (Calc.) (MBAR)**	0.87 (0.85)	0.76
**Exp vs Schrödinger (Calc.)**	0.86	0.74
**GROMACS (Calc.) (MBAR) vs Schrödinger (Calc.)**	0.93 (0.82)	0.86
**Schrödinger or GROMACS (previously reported) vs Schrödinger (Calc.)**	0.87	0.77

KENDALL_TAU	
**0.54**	Exp vs GROMACS (Calc.)
**0.62**	Exp vs Schrödinger (Calc.)
**0.63**	GROMACS (Calc.) vs Schrödinger (Calc.)
**0.76**	Schrödinger or GROMACS (previously reported) vs Schrödinger (Calc.)
**0.50**	Exp vs GROMACS (Calc.) MBAR
**0.58**	GROMACS (Calc.) MBAR vs Schrödinger (Calc.)

References

1. Duan, J., Lupyan, D. & Wang, L. Improving the Accuracy of Protein Thermostability Predictions for Single Point Mutations. *Biophys. J.* **119**, 115–127 (2020).
2. Werner, M., Gapsys, V. & de Groot, B. L. One Plus One Makes Three: Triangular Coupling of Correlated Amino Acid Mutations. *J. Phys. Chem. Lett.* **12**, 3195–3201 (2021).
