# Supplementary material for: Benchmarking free energy calculations: Analysis of single and double mutations across two simulation software platforms for two protein systems
Source: PLoS One. 2026 Apr 3;21(4):e0335829. doi: 10.1371/journal.pone.0335829 (PMC13048485; doi:10.1371/journal.pone.0335829)
Supplement: S1 Fig — Pearson correlation coefficient (r) and coefficient of determination (R²) are indicated. (PDF) [file pone.0335829.s002.pdf]

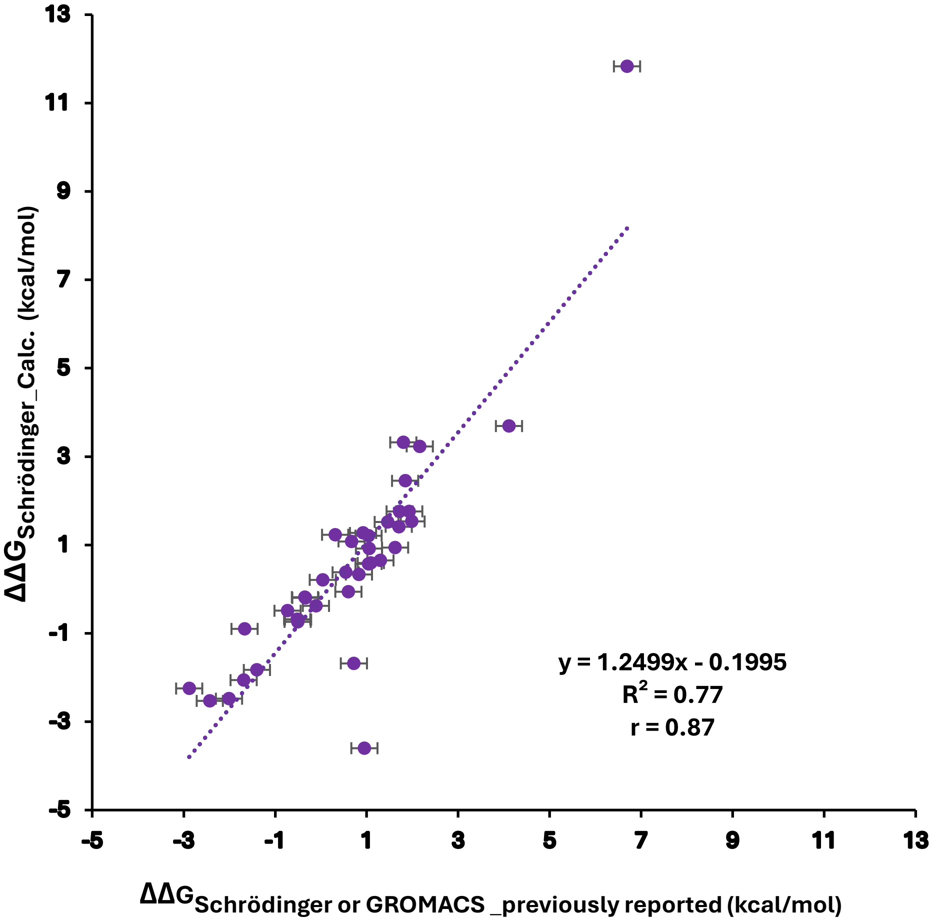

S1 Fig. Correlation plot of free energy changes ($\Delta\Delta G$) for 38 SMs in the S. nuclease protein, comparing previously reported values using Schrödinger or GROMACS with values calculated in this study using Schrödinger. Pearson correlation coefficient (r) and coefficient of determination (R^2) are indicated.
