# Supplementary material for: Benchmarking free energy calculations: Analysis of single and double mutations across two simulation software platforms for two protein systems
Source: PLoS One. 2026 Apr 3;21(4):e0335829. doi: 10.1371/journal.pone.0335829 (PMC13048485; doi:10.1371/journal.pone.0335829)
Supplement: S2 Table — The experimental values and previously reported values using GROMACS alongside values calculated in this study using Schrödinger are shown for comparison. (PDF) [file pone.0335829.s003.pdf]

S2 Table. Folding free energy changes ($\Delta\Delta G_{WT}^{AB}$) in kcal/mol for 45 double mutants (DMs) of the S. nuclease protein. The experimental values and previously reported values using GROMACS alongside values calculated in this study using Schrödinger are shown for comparison.

S. No.	DMs from S. nuclease	$\Delta\Delta G_{WT}^{AB}$ Exp[2]	$\Delta\Delta G_{WT}^{AB}$ GROMACS (previously reported)[2]	$\Delta\Delta G_{WT}^{AB}$ Schrödinger (Calc.)
1	**L7A+I15V**	2.07	1.40	0.73 ± 0.09
2	**L7A+V23F**	3.64	2.66	1.43 ± 0.18
3	**L7A+I72V**	2.40	2.02	1.34 ± 0.09
4	**L7A+L37A**	1.65	1.24	0.58 ± 0.17
5	**L7A+G79S**	2.76	1.12	1.21 ± 0.18
6	**L7A+Y85A**	1.60	0.54	1.35 ± 0.10
7	**L7A+I92V**	1.54	1.99	0.78 ± 0.09
8	**L7A+Y113A**	1.10	0.32	-0.49 ± 0.10
9	**L7A+A130G**	2.45	1.80	1.51 ± 0.09
10	**I15V+I72V**	2.00	3.17	1.81 ± 0.09
11	**I15V+Y85A**	1.06	1.78	1.92 ± 0.09
12	**I15V+Y113A**	0.62	1.15	0.32 ± 0.09
13	**I18M+T33S**	1.99	-0.69	0.29 ± 0.10
14	**I18M+A90S**	2.69	0.95	2.47 ± 0.09
15	**V23F+I15V**	2.58	2.55	2.21 ± 0.17
16	**V23F+L37A**	3.16	2.89	2.38 ± 0.21
17	**V23F+T33S**	2.71	2.22	2.57 ± 0.19
18	**V23F+A69T**	3.99	2.86	3.77 ± 0.13

19	**V23F+I72V**	2.89	3.03	2.73 ± 0.15
20	**V23F+G79S**	4.74	3.03	3.68 ± 0.19
21	**V23F+Y85A**	2.50	2.06	3.01 ± 0.14
22	**V23F+A90S**	3.30	3.74	4.58 ± 0.15
23	**V23F+I92V**	2.00	2.12	2.32 ± 0.15
24	**V23F+Y113A**	1.88	1.55	1.20 ± 0.17
25	**V23F+A130G**	3.49	2.93	3.10 ± 0.17
26	**T33S+A90S**	3.15	2.52	5.00 ± 0.08
27	**L37A+I15V**	2.16	2.08	1.97 ± 0.12
28	**L37A+T33S**	2.20	1.90	2.08 ± 0.16
29	**L37A+A69T**	4.12	3.89	3.94 ± 0.12
30	**L37A+I72V**	2.77	2.50	2.41 ± 0.15
31	**L37A+G79S**	1.82	1.50	1.61 ± 0.39
32	**L37A+Y85A**	1.97	2.36	2.50 ± 0.16
33	**L37A+A90S**	3.31	3.11	4.88 ± 0.13
34	**L37A+I92V**	1.76	2.70	2.24 ± 0.14
35	**L37A+Y113A**	1.45	1.39	1.06 ± 0.16
36	**L37A+A130G**	2.52	2.50	2.61 ± 0.13
37	**I72V+Y85A**	1.60	1.75	2.48 ± 0.09
38	**I72V+Y113A**	0.94	0.94	0.90 ± 0.10
39	**G79S+I15V**	3.09	2.36	2.65 ± 0.16
40	**G79S+I72V**	3.60	2.80	3.73 ± 0.17

41	**G79S+Y85A**	2.68	1.00	3.39 ± 0.18
42	**G79S+I92V**	2.54	2.17	2.95 ± 0.16
43	**G79S+Y113A**	2.21	0.70	1.26 ± 0.18
44	**G79S+A130G**	3.67	2.36	3.67 ± 0.19
45	**Y85A+Y113A**	0.23	0.39	0.89 ± 0.10
	**RMSE**		0.13	0.13

**Pearson correlation for all 45 DMs**	**Pearson r**	**R^2**
**Exp vs GROMACS (previously reported)[2]**	0.71	0.51
**Exp vs Schrödinger (Calc.)**	0.74	0.55
**GROMACS (previously reported)[2] vs Schrödinger (Calc.)**	0.71	0.51

KENDALL-TAU	
**0.52**	Exp vs Gromacs
**0.57**	Exp vs Schrodinger
**0.55**	Gromacs vs Schrodinger
