# Supplementary material for: Benchmarking free energy calculations: Analysis of single and double mutations across two simulation software platforms for two protein systems
Source: PLoS One. 2026 Apr 3;21(4):e0335829. doi: 10.1371/journal.pone.0335829 (PMC13048485; doi:10.1371/journal.pone.0335829)
Supplement: S3 Table — The experimental data and previously reported GROMACS values are presented alongside values calculated in this study using Schrödinger for comparative analysis. (PDF) [file pone.0335829.s005.pdf]

S3 Table. Nonadditivity values *(δ_{WT}^{AB})* in kcal/mol for 45 double mutants (DMs) of the S. nuclease protein. The experimental data and previously reported GROMACS values are presented alongside values calculated in this study using Schrödinger for comparative analysis.

**S. No.**	**DMs from S. nuclease**	δ_{WT}^{AB}**Exp**[2]	δ_{WT}^{AB}**GROMACS (previously reported)**[2]	δ_{WT}^{AB}**Schrödinger (Calc.)**
1	**L7A +I15V**	-0.31	-0.52	0.15 ± 0.10
2	**L7A +V23F**	-0.23	0.04	-0.27 ± 0.16
3	**L7A +L37A**	-1.60	-0.77	-0.77 ± 0.20
4	**L7A +I72V**	-0.94	-0.58	-0.13 ± 0.11
5	**L7A +G79S**	-1.47	-1.22	-1.18 ± 0.20
6	**L7A +Y85A**	-0.39	-0.35	0.18 ± 0.13
7	**L7A +I92V**	-0.54	-0.23	-0.04 ± 0.10
8	**L7A +Y113A**	-0.45	-0.16	-0.05 ± 0.11
9	**L7A +A130G**	-0.23	-0.20	0.05 ± 0.10
10	**I15V +I72V**	-0.57	-0.10	-0.36 ± 0.08
11	**I15V +Y85A**	-0.16	0.06	0.05 ± 0.11
12	**I15V +Y113A**	-0.16	-0.05	0.06 ± 0.10
13	**I18M +T33S**	0.50	0.18	0.11 ± 0.44
14	**I18M +A90S**	0.30	0.80	0.14 ± 0.12
15	**V23F +I15V**	-0.52	-0.49	-0.19 ± 0.15
16	**V23F +L37A**	-0.81	-1.02	-0.79 ± 0.24
17	**V23F +T33S**	-0.58	-0.37	-0.27 ± 0.45

18	**V23F +A69T**	-0.93	-1.22	-1.22 ± 0.98
19	**V23F +I72V**	-1.17	-0.85	-0.56 ± 0.15
20	**V23F +G79S**	-0.21	-0.96	-0.53 ± 0.22
21	**V23F +Y85A**	-0.20	-0.10	0.02 ± 0.15
22	**V23F +A90S**	-0.89	0.10	-0.41 ± 0.15
23	**V23F +I92V**	-0.79	-1.52	-0.32 ± 0.13
24	**V23F +Y113A**	-0.38	-0.35	-0.18 ± 0.14
25	**V23F +A130G**	0.09	-0.33	-0.18 ± 0.13
26	**T33S +A90S**	0.26	0.01	0.69 ± 0.44
27	**L37A +I15V**	-0.32	-1.01	-0.08 ± 0.18
28	**L37A +T33S**	-0.47	-0.36	-0.41 ± 0.47
29	**L37A +A69T**	-0.18	0.05	-0.70 ± 0.98
30	**L37A +I72V**	-0.67	-1.19	-0.53 ± 0.18
31	**L37A +G79S**	-2.50	-1.63	-2.25 ± 0.35
32	**L37A +Y85A**	-0.11	0.33	-0.14 ± 0.20
33	**L37A +A90S**	-0.26	-0.39	0.24 ± 0.19
34	**L37A +I92V**	-0.41	-0.59	-0.05 ± 0.18
35	**L37A +Y113A**	-0.19	-0.35	0.03 ± 0.19
36	**L37A +A130G**	-0.26	-0.64	-0.32 ± 0.18
37	**I72V +Y85A**	-0.58	-0.45	-0.28 ± 0.11
38	**I72V +Y113A**	-0.80	-0.80	-0.25 ± 0.09
39	**G79S +I15V**	-0.37	-0.83	-0.44 ± 0.19

40	**G79S +I72V**	-0.82	-1.12	-0.25 ± 0.18
41	**G79S +Y85A**	-0.38	-1.16	-0.29 ± 0.20
42	**G79S +I92V**	-0.61	-1.36	-0.38 ± 0.17
43	**G79S +Y113A**	-0.41	-0.96	-0.81 ± 0.20
44	**G79S +A130G**	-0.09	-0.94	-0.30 ± 0.19
45	**Y85A +Y113A**	-0.15	0.15	0.04 ± 0.12
	**RMSE**		0.07	0.06

**Pearson correlation for all 45 DMs cycle (nonadditivity)**	**Pearson r**	**R^2**
**Exp vs GROMACS (previously reported)[2]**	0.63	0.40
**Exp vs Schrödinger (Calc.)**	0.79	0.63
**GROMACS (previously reported)[2] vs Schrödinger (Calc.)**	0.61	0.38

**KENDALL_TAU**	
**0.48**	Exp vs Gromacs
**0.44**	Exp vs Schrodinger
**0.45**	Gromacs vs Schrodinger
