# Supplementary material for: Benchmarking free energy calculations: Analysis of single and double mutations across two simulation software platforms for two protein systems
Source: PLoS One. 2026 Apr 3;21(4):e0335829. doi: 10.1371/journal.pone.0335829 (PMC13048485; doi:10.1371/journal.pone.0335829)
Supplement: S3 Fig — Pearson correlation coefficient (r) and coefficient of determination (R²) are shown. (PDF) [file pone.0335829.s006.pdf]

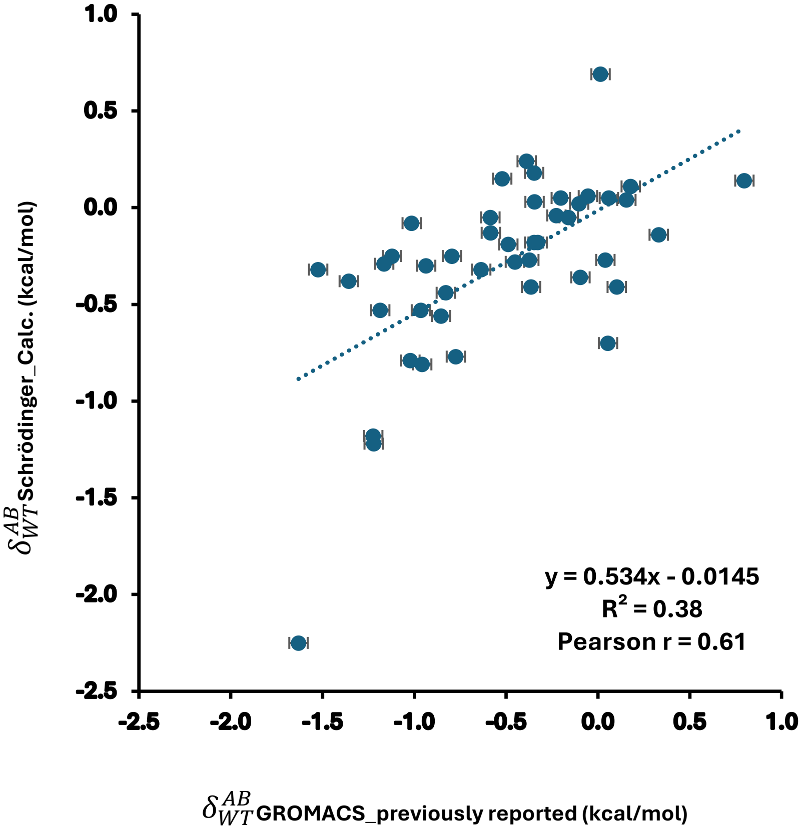

S3 Fig. Correlation plot of nonadditivity (δ_{WT}^{AB}) for 45 DMs in the S. nuclease protein, comparing previously reported values from GROMACS with values calculated in this study using Schrödinger. Pearson correlation coefficient (r) and coefficient of determination (R^2) are shown.
