# Supplementary material for: Benchmarking free energy calculations: Analysis of single and double mutations across two simulation software platforms for two protein systems
Source: PLoS One. 2026 Apr 3;21(4):e0335829. doi: 10.1371/journal.pone.0335829 (PMC13048485; doi:10.1371/journal.pone.0335829)
Supplement: S4 Table — The experimental values and previously reported values using Schrödinger alongside values calculated in this study using GROMACS and Schrӧdinger are provided for comparison. (PDF) [file pone.0335829.s007.pdf]

S4 Table. Folding free energy changes ($\Delta\Delta G$) in kcal/mol for 24 single mutants (SMs) of the T4 lysozyme protein. The experimental values and previously reported values using Schrödinger alongside values calculated in this study using GROMACS and Schrödinger are provided for comparison.

**S. No.**	**SMs from T4 lysozyme**	$\Delta\Delta G_{Exp}$[1]	$\Delta\Delta G_{Schrödinger}$ **(previously reported)**[1]	$\Delta\Delta G_{GROMACS}$ **(Calc.)**	$\Delta\Delta G_{Schrödinger}$ **(Calc.)**	**GROMACS (Calc.) Using MBAR**
1	**I3Y**	2.3	3.07	1.2 ± 0.41	3.31 ± 0.43	1.12 ± 0.04
2	**I3V**	0.4	0.8	0.89 ± 0.20	1.1 ± 0.41	0.83 ± 0.01
3	**M6I**	1.4	2.95	1.14 ± 0.39	2.83 ± 0.41	0.8 ± 0.01
4	**N55G**	0.6	0.87	0.48 ± 0.30	0.42 ± 0.41	0.73 ± 0.04
5	**G77A**	-0.4	-1.5	-0.9 ± 0.22	-0.98 ± 0.41	-0.52 ± 0.01
6	**A82P**	-0.8	-1.33	NA	-1.25 ± 0.41	NA
7	**G113A**	-0.3	-0.68	-0.78 ± 0.13	-1.06 ± 0.41	-0.47 ± 0.01
8	**T115E**	-0.3	-1.95	0.03 ± 0.84	-1.34 ± 0.42	0.22 ± 0.12
9	**Q123E**	-0.4	-1.09	-0.13 ± 0.35	-0.06 ± 0.41	-0.58 ± 0.28
10	**K124G**	0.1	2.14	0.85 ± 0.25	1.91 ± 0.42	1.05 ± 0.09
11	**S38N**	0	-0.34	-0.72 ± 0.23	0.05 ± 0.41	-0.77 ± 0.04
12	**S44A**	-0.3	-0.11	-0.14 ± 0.16	-0.35 ± 0.41	-0.14 ± 0.01
13	**L46A**	1.9	1.82	2.08 ± 0.60	2.14 ± 0.41	1.82 ± 0.03
14	**D47A**	1	0.32	1.22 ± 0.84	1.16 ± 0.41	1.33 ± 0.07
15	**T59A**	1.5	1.46	0.9 ± 0.23	0.94 ± 0.41	0.9 ± 0.01
16	**T59N**	1.1	0.09	0.64 ± 0.74	0.42 ± 0.41	0.47 ± 0.05

17	**T59D**	1.2	2.77	2.09 ± 0.99	2.05 ± 0.43	2.04 ± 0.09
18	**T59G**	1.6	0.18	0.63 ± 0.44	0.45 ± 0.41	0.88 ± 0.03
19	**T59S**	0.2	0.32	0.16 ± 0.35	0.13 ± 0.42	0.22 ± 0.02
20	**T59V**	1.5	2.9	2.46 ± 0.35	2.31 ± 0.46	2.46 ± 0.02
21	**D92N**	1.4	3.18	2.41 ± 0.06	2.5 ± 0.42	2.12 ± 0.24
22	**T109N**	-0.1	0.11	-0.23 ± 0.76	-0.15 ± 0.42	-0.15 ± 0.04
23	**T109D**	-0.6	-0.33	-1.37 ± 0.45	-0.3 ± 0.41	-1.27 ± 0.07
24	**N144E**	-0.5	-1.35	0.39 ± 0.29	-1.15 ± 0.11	0.36 ± 0.11
	**RMSE**			0.13	0.16	0.13

**Pearson correlation for all 24 SMs**	**Pearson r**	**R^2**
**Exp vs GROMACS (Calc.) (MBAR)**	0.80 (0.78)	0.63
**Exp vs Schrödinger (Calc.)**	0.85	0.72
**GROMACS (Calc.) (MBAR) vs Schrödinger (Calc.)**	0.81 (0.77)	0.65
**Schrödinger (previously reported)[1] vs Schrödinger (Calc.)**	0.96	0.92

KENDALL_TAU	
**0.61**	Exp vs GROMACS (Calc.)
**0.68**	Exp vs Schrödinger (Calc.)
**0.68**	GROMACS (Calc.) vs Schrödinger (Calc.)
**0.81**	Schrödinger (previously reported)1 vs Schrödinger (Calc.)
**0.61**	Exp vs GROMACS (Calc.) MBAR
**0.61**	GROMACS (Calc.) MBAR vs Schrödinger (Calc.)
