# Supplementary material for: Benchmarking free energy calculations: Analysis of single and double mutations across two simulation software platforms for two protein systems
Source: PLoS One. 2026 Apr 3;21(4):e0335829. doi: 10.1371/journal.pone.0335829 (PMC13048485; doi:10.1371/journal.pone.0335829)
Supplement: S4 Fig — Pearson correlation coefficient (r) and coefficient of determination (R²) are shown. (PDF) [file pone.0335829.s008.pdf]

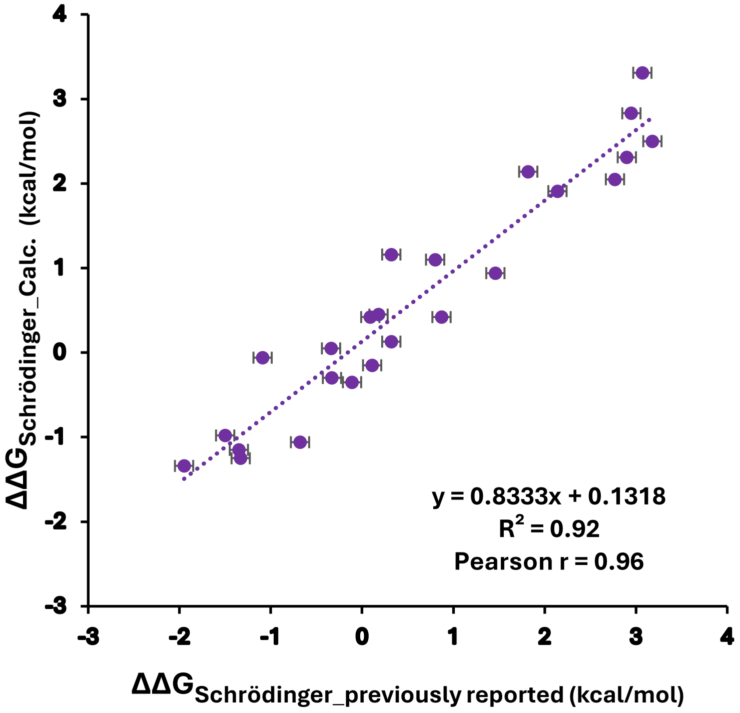

S4 Fig. Correlation plot of free energy changes ($\Delta\Delta G$) for 24 SMs in the T4 lysozyme protein, comparing previously reported values with calculated values from Schrödinger. Pearson correlation coefficient (r) and coefficient of determination (R^2) are shown.
