# Supplementary material for: Benchmarking free energy calculations: Analysis of single and double mutations across two simulation software platforms for two protein systems
Source: PLoS One. 2026 Apr 3;21(4):e0335829. doi: 10.1371/journal.pone.0335829 (PMC13048485; doi:10.1371/journal.pone.0335829)
Supplement: S5 Fig — Each of the four Figs consists of two panels: the upper panel displays the overlap for the tripeptide, while the lower panel displays the overlap for the full protein system. (PDF) [file pone.0335829.s009.pdf]

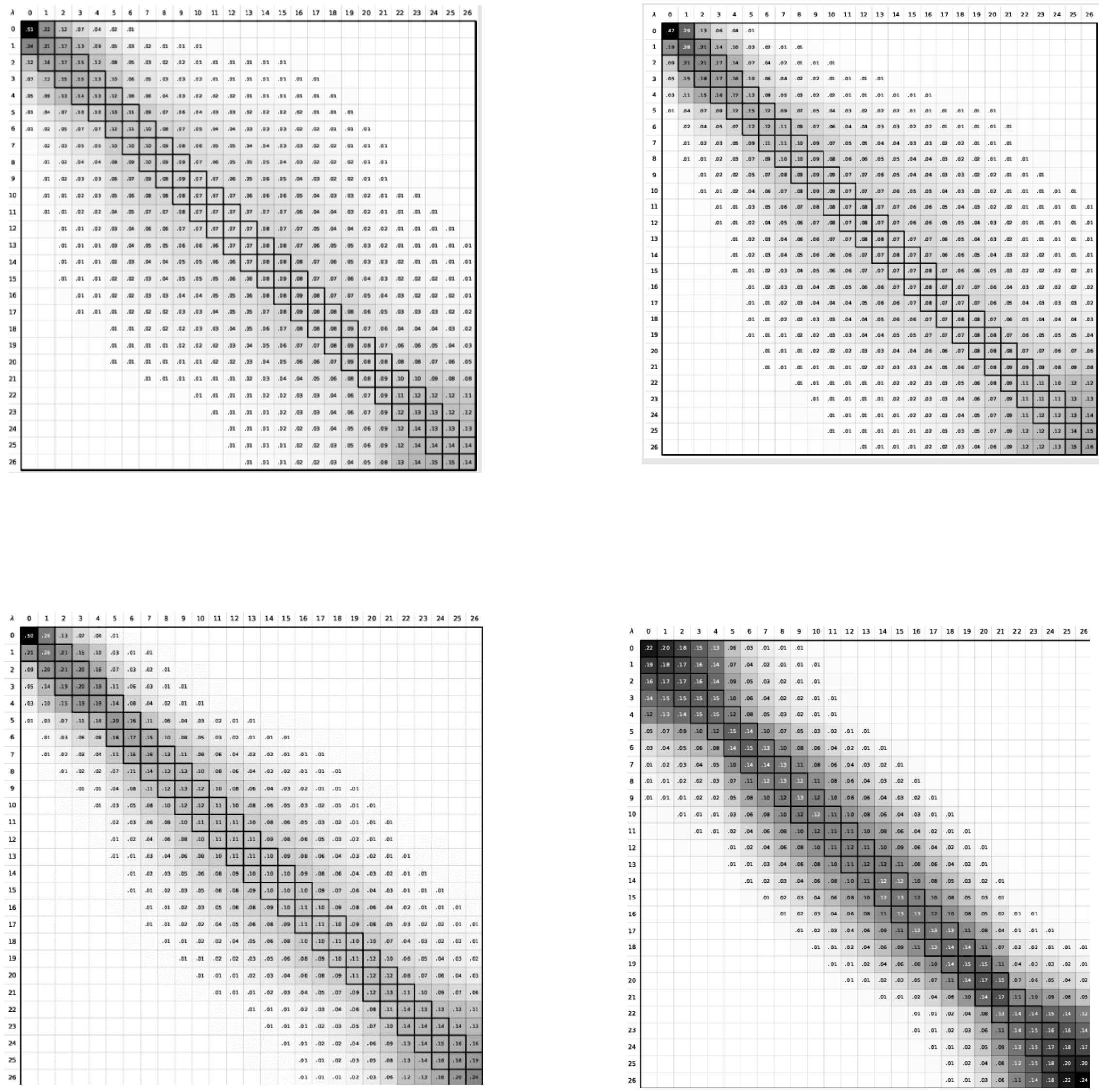

S5 Fig. Overlap matrix plots for representative *S. nuclease* mutations (T41C and T41V) with all first off-diagonal entries well above 0.03, the suggested threshold. Each of the four Figs consists of two panels: the upper panel displays the overlap for the tripeptide, while the lower panel displays the overlap for the full protein system.
