# Supplementary material for: Benchmarking free energy calculations: Analysis of single and double mutations across two simulation software platforms for two protein systems
Source: PLoS One. 2026 Apr 3;21(4):e0335829. doi: 10.1371/journal.pone.0335829 (PMC13048485; doi:10.1371/journal.pone.0335829)
Supplement: S8 Fig — The plots show ΔΔG versus simulation time across four Figs. In each case, the top panel corresponds to the tripeptide simulation (unfolded state proxy) and the bottom panel corresponds to the full protein simulation (folded state). (PDF) [file pone.0335829.s012.pdf]

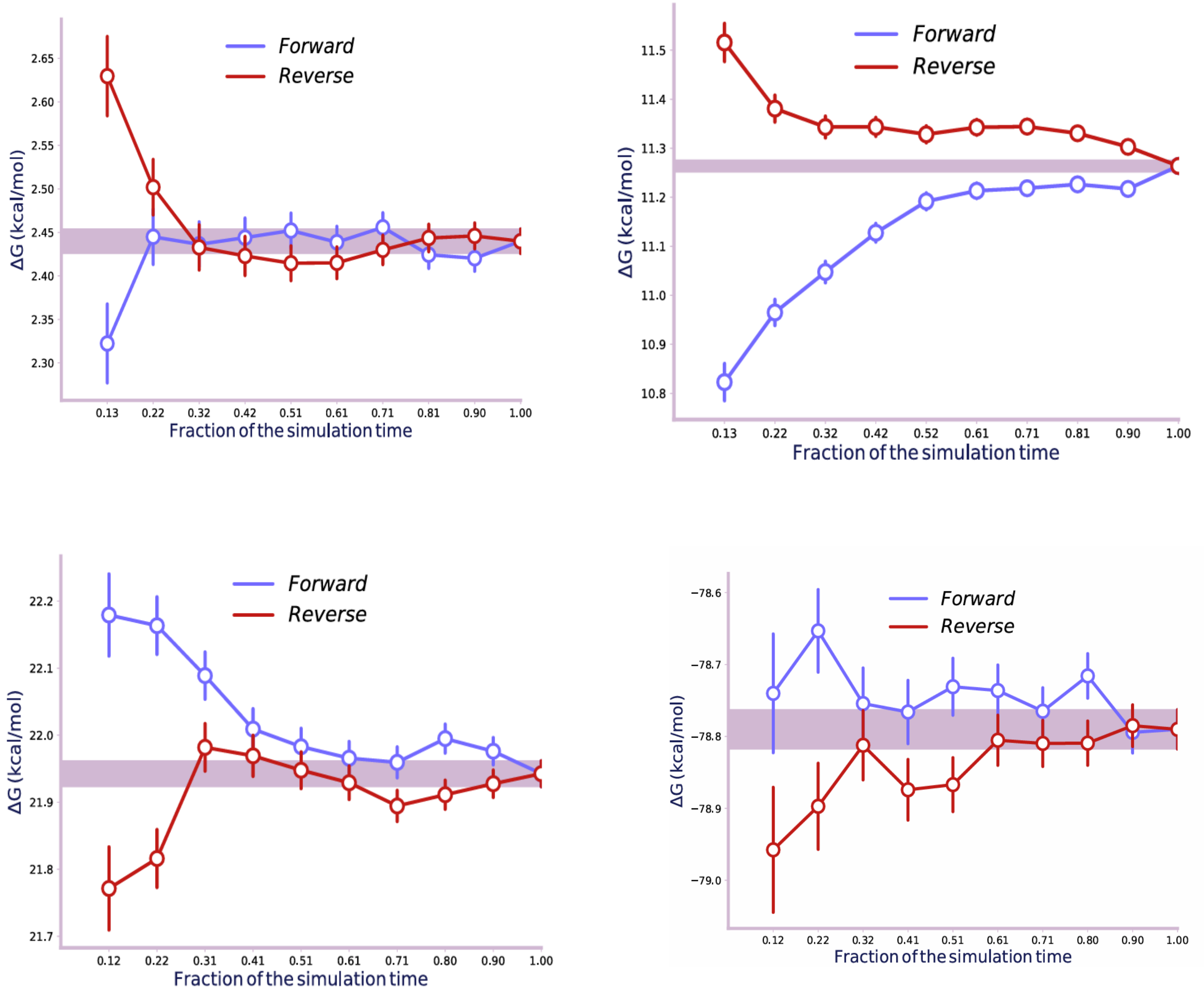

S8 Fig. Free energy convergence for representative T4 lysozyme mutants S44A and D47A. The plots show ΔΔG versus simulation time across four Figs. In each case, the top panel corresponds to the tripeptide simulation (unfolded state proxy) and the bottom panel corresponds to the full protein simulation (folded state).
