# Supplementary material for: Benchmarking free energy calculations: Analysis of single and double mutations across two simulation software platforms for two protein systems
Source: PLoS One. 2026 Apr 3;21(4):e0335829. doi: 10.1371/journal.pone.0335829 (PMC13048485; doi:10.1371/journal.pone.0335829)
Supplement: S5 Table — The mutations are categorized by charge, size, and location within the protein structure. (PDF) [file pone.0335829.s013.pdf]

S5 Table. Calculated free energy changes (kcal/mol) for 38 single mutants (SMs) of S. nuclease using Schrödinger and GROMACS, compared against experimental values. The mutations are categorized by charge, size, and location within the protein structure.

| S.No | Staph Nuclease | Charged or Neutral | Size | Mutation Location | $\Delta\Delta G_{exp} - \Delta\Delta G_{GROMACS}$ | $\Delta\Delta G_{exp} - \Delta\Delta G_{Schrödinger}$ |
|---|---|---|---|---|---|---|
| 1 | T22C | Neutral | NC | Buried | 0.34 | 1.08 |
| 2 | T22V | Neutral | ↑ | Buried | -0.18 | -0.02 |
| 3 | V23L | Neutral | ↑ | Buried | 0.34 | 0.3 |
| 4 | L25I | Neutral | NC | Buried | 0.52 | -0.06 |
| 5 | T33V | Neutral | ↑ | Buried | -0.46 | 0.34 |
| 6 | T41C | Neutral | NC | Buried | -0.09 | 0.08 |
| 7 | T41I | Neutral | ↑ | Buried | 0.38 | 1.13 |
| 8 | T41S | Neutral | NC | Surface | 0.26 | -0.11 |
| 9 | T41V | Neutral | ↑ | Buried | -0.01 | 1.26 |
| 10 | T44V | Neutral | ↑ | Buried | -0.94 | -0.31 |
| 11 | S59A | Neutral | ↓ | Surface | -0.18 | -0.01 |
| 12 | T62S | Neutral | NC | Surface | 1.54 | 1.51 |
| 13 | T62V | Neutral | ↑ | Surface | 0.39 | 2.73 |
| 14 | V66I | Neutral | ↑ | Buried | 0.56 | 1.69 |
| 15 | I72L | Neutral | NC | Buried | -0.44 | -0.18 |
| 16 | T82S | Neutral | NC | Surface | -0.13 | -0.57 |
| 17 | I92V | Neutral | ↓ | Buried | -0.94 | -0.17 |
| 18 | K116G | Charged | ↓ | Surface | -0.6 | 1.25 |
| 19 | T120C | Neutral | NC | Surface | 0.82 | 0.76 |
| 20 | T120S | Neutral | NC | Surface | 0.64 | 0.27 |
| 21 | T120V | Neutral | ↑ | Surface | 0.57 | -1.89 |
| 22 | S128A | Neutral | ↓ | Surface | 0.31 | 1.78 |
| 23 | V66K | Charged | ↑ | Surface | -0.09 | -4.33 |
| 24 | L7A | Neutral | ↓ | Buried | 1.76 | 1.65 |
| 25 | I15V | Neutral | ↓ | Buried | -0.42 | 0.16 |
| 26 | I18M | Neutral | NC | Buried | 0.95 | 1.4 |
| 27 | V23F | Neutral | ↑ | Buried | 1.62 | 0.55 |
| 28 | T33S | Neutral | NC | Buried | -0.15 | -0.08 |

| | | | | | | |
|---|---|---|---|---|---|---|
| 29 | L37A | Neutral | ↓ | Buried | 0.28 | 0.28 |
| 30 | T62A | Neutral | ↓ | Surface | 1.32 | 4.09 |
| 31 | V66L | Neutral | ↑ | Buried | 1.56 | 3.7 |
| 32 | A69T | Neutral | ↑ | Buried | 0.35 | -0.59 |
| 33 | I72V | Neutral | ↓ | Buried | 0.25 | 0.25 |
| 34 | G79S | Neutral | ↑ | Buried | 2.14 | 0.22 |
| 35 | Y85A | Neutral | ↓ | Buried | -0.23 | -0.82 |
| 36 | A90S | Neutral | ↑ | Buried | -0.65 | -1.41 |
| 37 | Y113A | Neutral | ↓ | Surface | -0.51 | 0.35 |
| 38 | A130G | Neutral | NC | Surface | 0.07 | -0.4 |
