# Supplementary material for: Benchmarking free energy calculations: Analysis of single and double mutations across two simulation software platforms for two protein systems
Source: PLoS One. 2026 Apr 3;21(4):e0335829. doi: 10.1371/journal.pone.0335829 (PMC13048485; doi:10.1371/journal.pone.0335829)
Supplement: S6 Table — The mutations are categorized by charge, size, and location within the protein structure. (PDF) [file pone.0335829.s014.pdf]

S6 Table. Calculated free energy changes (kcal/mol) for 24 single mutants (SMs) of T4 lysozyme using Schrödinger and GROMACS, compared against experimental values. The mutations are categorized by charge, size, and location within the protein structure.

S. No.	T4 lysozyme	Charged or Neutral	Size	Mutation Location	$\Delta\Delta G_{exp} - \Delta\Delta G_{GROMACS}$	$\Delta\Delta G_{exp} - \Delta\Delta G_{Schrödinger}$
1	I3Y	Neutral	↑	Buried	1.1	-1.01
2	I3V	Neutral	↓	Buried	-0.49	-0.7
3	M6I	Neutral	NC	Buried	0.26	-1.43
4	N55G	Neutral	↓	Buried	0.12	0.18
5	G77A	Neutral	NC	Buried	0.5	0.58
6	A82P	Neutral	↑	Buried	NA	0.45
7	G113A	Neutral	NC	Buried	0.48	0.76
8	T115E	Charged	↑	Surface	-0.33	1.04
9	Q123E	Charged	NC	Surface	-0.27	-0.34
10	K124G	Charged	↓	Surface	-0.75	-1.81
11	S38N	Neutral	↑	Surface	0.72	-0.05
12	S44A	Neutral	↓	Surface	-0.16	0.05
13	L46A	Neutral	↓	Buried	-0.18	-0.24
14	D47A	Charged	↓	Buried	-0.22	-0.16
15	T59A	Neutral	↓	Buried	0.6	0.56
16	T59N	Neutral	↑	Surface	0.46	0.68
17	T59D	Charged	↑	Surface	-0.89	-0.85
18	T59G	Neutral	↓	Buried	0.97	1.15
19	T59S	Neutral	NC	Surface	0.04	0.07
20	T59V	Neutral	↑	Buried	-0.96	-0.81
21	D92N	Charged	NC	Buried	-1.01	-1.1
22	T109N	Neutral	↑	Surface	0.13	0.05
23	T109D	Charged	↑	Surface	0.77	-0.3
24	N144E	Charged	↑	Surface	-0.89	0.65
