# Supplementary material for: Benchmarking free energy calculations: Analysis of single and double mutations across two simulation software platforms for two protein systems
Source: PLoS One. 2026 Apr 3;21(4):e0335829. doi: 10.1371/journal.pone.0335829 (PMC13048485; doi:10.1371/journal.pone.0335829)
Supplement: Attachment — Submitted filename: review.docx [file pone.0335829.s015.docx]

The paper is about the benchmark of mutation ddG on two software platforms (FEP+ and Gromacs), on 2 protein system.

1. Correlation should not be the main matrix of comparison. RMSE, kendall tau, or AUE (already being used in the results and discussion), can also be used to evaluate the quality of free energy calculations. And Correlation of ddG is not well defined, because of the following reason. There is not really a direction of A to B and B to A, but plotting some ddG edges of A to B or B to A will give different correlation analysis results.
2. In the method section of “FEP calculations using GROMACS”. Please more clearly explain: 1. What soft-core potential is used? Beutler or Gapsys 2. Soft-core potential is applied to both vdw and coulomb or vdw only. 3. “from λ= 0 to λ = 26 …” is confusing and inconsistent with equation 4 in which λ ϵ [0,1]. 4. Are those 27 windows simulated separately or together with Hamiltonian replica exchange. Hamiltonian replica exchange is an available enhanced sampling method in the native Gromacs mdrun. It does not affect simulation through put while bringing in sampling enhancement. If not using, please justify the reason for not using. 5. “AMBER99sb*ILDN” was stated in method, but “Amber99SB-ILDN” was stated in the abstract. 6. How was the charge neutralized when a charge mutation was applied? 7. Which ion parameters were used? 8. Please clarify the exact lambda schedule for example: 0.0, 0.05, … 1.00 for electrostatic … 9. As a benchmark, all the input files (structure, Gromacs mdp files for every steps), analysis files, and results files (e.i. ddG values in csv file) should be provided, either in SI or a openly available repository. Not only should the value of free energy estimation be reported, the uncertainties of each free energy estimation should also be reported.
3. Please make the x-y axis aspect equal when comparing experiment and computed delta G. As 1 kcal/mol in the experiment means the same as 1 kcal/mol in the experiment.
4. Two platforms (FEP+ and gromacs) are using different force fields, enhanced sampling algorithms, and sampling time. From the benchmark results, can we get some insight about the advantages and disadvantages about OPLS4 vs amber99*ildn and REST2 with 16/24 lambda vs 27 lambda with no replica exchange?
5. Does the magnitude of the error correlate with the type of mutation (sides of the amino acid, neutral or charged mutation). Does the error correlate with the place of the mutation (the surface of the protein or in the core of the protein.) With this benchmark, can we get some insight about what kind of mutation is more difficult to calculate with alchemical free energy calculations.
